# Variability in Criteria for Emergency Medical Services Routing of Acute Stroke Patients to Designated Stroke Center Hospitals

**DOI:** 10.5811/westjem.2015.7.26179

**Published:** 2015-10-20

**Authors:** Nikolay Dimitrov, William Koenig, Nichole Bosson, Sarah Song, Jeffrey L. Saver, William J. Mack, Nerses Sanossian

**Affiliations:** * University of Southern California, Keck School of Medicine, Los Angeles, California; † University of Southern California, Keck School of Medicine, Department of Neurology, Los Angeles, California; ‡ University of Southern California, Keck School of Medicine, Department of Neurosurgery, Los Angeles, California; § University of Southern California, Keck School of Medicine, Roxanna Todd Hodges Comprehensive Stroke Clinic, Los Angeles, California; ¶ Los Angeles County Emergency Medical Services Agency, Los Angeles, California; || Rush University, Department of Neurology, Chicago, Illinois; # University of California, Los Angeles Stroke Center, Los Angeles, California

## Abstract

**Introduction:**

Comprehensive stroke systems of care include routing to the nearest designated stroke center hospital, bypassing non-designated hospitals. Routing protocols are implemented at the state or county level and vary in qualification criteria and determination of destination hospital. We surveyed all counties in the state of California for presence and characteristics of their prehospital stroke routing protocols.

**Methods:**

Each county’s local emergency medical services agency (LEMSA) was queried for the presence of a stroke routing protocol. We reviewed these protocols for method of stroke identification and criteria for patient transport to a stroke center.

**Results:**

Thirty-three LEMSAs serve 58 counties in California with populations ranging from 1,175 to nearly 10 million. Fifteen LEMSAs (45%) had stroke routing protocols, covering 23 counties (40%) and 68% of the state population. Counties with protocols had higher population density (1,500 vs. 140 persons per square mile). In the six counties without designated stroke centers, patients meeting criteria were transported out of county. Stroke identification in the field was achieved using the Cincinnati Prehospital Stroke Screen in 72%, Los Angeles Prehospital Stroke Screen in 7% and a county-specific protocol in 22%.

**Conclusion:**

California EMS prehospital acute stroke routing protocols cover 68% of the state population and vary in characteristics including activation by symptom onset time and destination facility features, reflecting matching of system design to local geographic resources.

## INTRODUCTION

In an effort to improve care and reduce the morbidity and mortality caused by stroke, the American Heart Association (ASA) developed recommendations for the development of stroke systems for specialized stroke care. The ASA recommendations include adoption of emergency medical services (EMS) protocols for the identification and rapid transport of acute stroke patients to primary stroke centers (PSCs). Furthermore, it is recommended that EMS responders preliminarily notify the receiving hospital in order to alert the hospital-based acute stroke team of the incoming patient.^2^ Thus, stroke systems are designed to streamline recognition, transport and initiation of care for acute stroke by establishing policies for preferentially routing stroke patients to designated stroke centers.

An increasing number of regions of the U.S. have adopted EMS stroke routing protocols since 2000.^1^–^2^ Beginning with counties in Alabama and Texas, policies for routing acute stroke patients to primary stroke centers were in place in 16 states by 2010, covering 53% of the U.S. population.^1^ Routing policies are determined on a county or state level and differ based on the needs and infrastructures of the regions they cover. Thus, a considerable variation exists between the parameters that determine conditions for initiation of routing in different regions across the country. Such parameters may include the following: maximum onset of stroke symptoms prior to transport or hospital arrival, criteria for detecting stroke cases by EMS responders, maximum routing time and a variety of others. We surveyed the counties of the state of California for acute stroke EMS routing policies and compared them based on the variables listed above.

## METHODS

We contacted the local EMS agency (LEMSA) office for each county in California to inquire about the presence of routing policies for stroke. If a routing policy was in place, we obtained a copy of the policy. Upon review of each policy, we obtained characteristics that included the following: maximum time from symptom onset to EMS evaluation to qualify for routing; type of stroke identification tool; and whether there is a maximum transportation time limit qualifier. We also looked at the number of hospitals in each county and their designation as either a primary or comprehensive stroke center. County and state population information was obtained using the 2010 census data.

## RESULTS

There were 33 LEMSAs serving 58 counties in California with populations ranging from 1,175 persons to nearly 10 million persons (mean 642,000, median 179,000). Counties varied in area ranging from 47 to 20,000 square mile (mean 2,690, median 1,540) and population density two to 17,000 persons per square mile (mean 661, median 104). Fifteen LEMSAs (45%) had acute stroke routing protocols, covering 23 counties (40%) and accounting for 68% of the overall state population (Table).

Counties with acute stroke routing protocols had higher population density (mean 1,500 vs. 140 persons per square mile, median 198 vs. 58 persons per square mile) compared to those without. All protocols designated a maximum time period from symptom onset to EMS evaluation to qualify for routing, but there was large variability ranging from two to eight hours, with a median of three hours (IQR 2.5–4) after symptom onset. Twelve of 23 (52%) allowed a maximum transport time of 30 minutes to qualify for diversion. In cases where transport time to the designated stroke center exceeded 30 minutes, patients would be routed to closest hospital. The median number of LEMSA-designated stroke hospitals per county in jurisdictions with routing was two (IQR 0–7, range 0–29). In the six counties without designated stroke centers, patients meeting criteria were transported out of county.

Regardless of the presence of a stroke routing policy, most LEMSAs (32 of 33, 97%) and counties (55 of 58, 95%) had designated a prehospital stroke identification instrument. LEMSA used the Cincinnati Prehospital Stroke Screen/Face Arm Speech Time (N=23, 72%), county-specific protocols (N=7, 22%) and Los Angeles Prehospital Stroke Screen (N=2, 7%).

## DISCUSSION

As of September 2013, 23 out of 58 California counties have implemented stroke routing policies, the first coming into effect in 2006 (Figure). These EMS prehospital acute stroke routing policies currently cover 68% of the state’s population. There are benefits of stroke routing policies in improving care, but also in increasing the numbers of hospitals seeking stroke center certification.^3^–^5^

One barrier to initiating these acute stroke routing protocols may be lack of appropriate facilities in scarcely populated regions. Of the 23 counties with routing policies, six transport patients to out-of-county PSCs, providing one possible solution to this problem. All counties with routing policies have designated stroke recognition criteria and set a maximum time of onset of symptoms prior to routing, as stipulated by the ASA in establishing stroke systems of care. Furthermore, 12 of these counties limited transport time to 30 minutes, meaning that if transport to a PSC was estimated to exceed 30 minutes, the patient would be taken to a closer, non-stroke-certified receiving facility. Variation in routing policies between different counties demonstrates the necessity of adapting stroke systems of care to the resources and infrastructures available in different regions.

Acute stroke routing is likely to benefit patients whose onset of symptoms falls within the time limit of eligibility for intravenous thrombolysis, between 3 and 4.5 hours.^5^–^6^ Thrombolysis, or acute stroke treatment, requires a synchronized and expeditious response to stroke emergencies involving prehospital, emergency department and hospital medical care. Well-trained first response personnel are required to identify potential stroke cases. Thus, all surveyed EMS routing protocols specify stroke recognition criteria to be used at the initial scene. If a stroke is suspected, the emergency responders must determine if the patient should be routed to the nearest designated stroke center instead of the nearest non-designated eligible facility. Protocols establish a straightforward method for making this decision by stipulating a maximum time for onset of symptoms prior to routing, and in some cases, limiting transport time. It is also necessary to alert the receiving facility of an incoming stroke case, in order to allow medical personnel to mobilize and prepare for potential acute stroke treatment.^8^ Routing protocols streamline this course of events and allow a more efficient response to stroke emergencies.^7^

Based on the finding of this study, 32% of California’s population does not have access to acute stroke routing. Future research should focus on establishing this figure on a national scale and determining the barriers that must be overcome in order to extend coverage to more people. Further work is also necessary to evaluate the difference in stroke patient outcomes between regions with and without stroke routing policies.

## Figures and Tables

**Figure:**
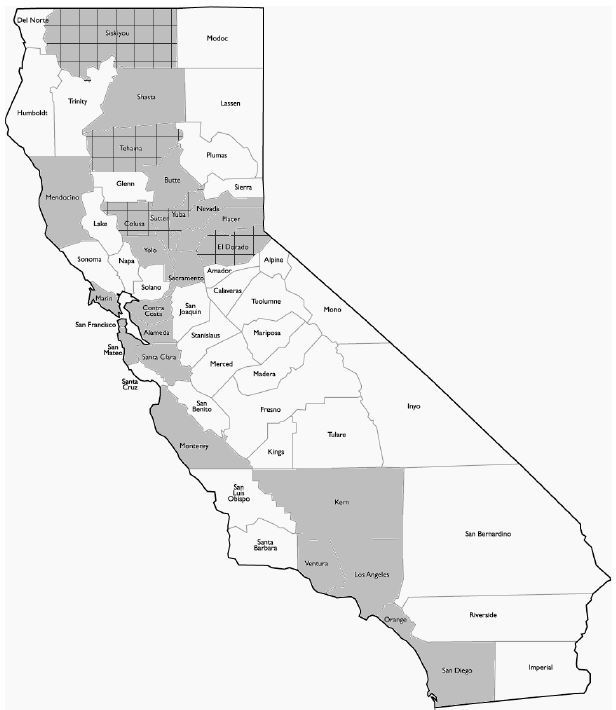
Map of California counties with emergency medical services stroke routing. Grey indicates all counties with routing policies as of September 2013. Counties that route out-of-county are indicated with a grid pattern.

**Table:** Number of counties and local emergency medicine services agencies (LEMSAs) fulfilling key stroke routing policies.

Stroke routing protocol	Number of counties	Number of LEMSAs
Yes	23	15
No	35	18
Stroke detection criteria		
CPSS	20	13
LAPSS	1	1
own protocol	2	1
Max time of onset of symptoms for routing		
2–3hrs	12	4
3.5–4.5hrs	8	8
5–8hrs	3	3
Maximum routing time		
30 min	12	4
not specified	11	11
Number of receiving PSCs in county		
0	6	
1–5	10	
6–10	6	
>10	1	

*CPSS,* Cincinnati prehospital stroke screen; *LAPSS*, Los Angeles prehospital stroke screen; *PSCs*, primary stroke centers
